# *Disrupted-in-schizophrenia-1* (DISC1) Regulates Endoplasmic Reticulum Calcium Dynamics

**DOI:** 10.1038/srep08694

**Published:** 2015-03-03

**Authors:** Sung Jin Park, Jaehoon Jeong, Young-Un Park, Kyung-Sun Park, Haeryun Lee, Namgyu Lee, Sung-Mo Kim, Keisuke Kuroda, Minh Dang Nguyen, Kozo Kaibuchi, Sang Ki Park

**Affiliations:** 1 Department of Life Sciences, Pohang University of Science and Technology, 790–784, Republic of Korea; 2 Division of Integrative Biosciences and Biotechnology, Pohang University of Science and Technology, 790–784, Republic of Korea; 3 Department of Cell Pharmacology, Nagoya University Graduate School of Medicine, Nagoya, Aichi 466–8550, Japan; 4 Hotchkiss Brain Institute, Departments of Clinical Neurosciences, Cell Biology and Anatomy, and Biochemistry and Molecular Biology, University of Calgary, Calgary, AB, Canada T2N 4N1

## Abstract

*Disrupted-in-schizophrenia-1* (DISC1) has emerged as a convincing susceptibility gene for multiple mental disorders, but its mechanistic link to the pathogenesis of schizophrenia related psychiatric conditions is yet to be further understood. Here, we showed that DISC1 localizes to the outer surface of the endoplasmic reticulum (ER). EXOC1, a subunit of the exocyst complex, interacted with DISC1 and affected its recruitment to inositol-1,4,5-trisphosphate receptor 1 (IP_3_R1). Notably, knockdown of DISC1 and EXOC1 elicited an exaggerated ER calcium response upon stimulation of IP_3_R agonists. Similar abnormal ER calcium responses were observed in hippocampal neurons from DISC1-deficient mutant mice. Moreover, perturbation of ER calcium dynamics upon DISC1 knockdown was effectively reversed by treatment with antipsychotic drugs, such as clozapine and haloperidol. These results collectively indicate that DISC1 is a regulatory factor in ER calcium dynamics, linking a perturbed intracellular calcium signaling and schizophrenia pathogenesis.

Schizophrenia is a mental disease characterized by a distorted perception of reality, commonly manifested in the form of hallucinations, delusions, or paranoia^1^. About 0.6–1% of the global population suffer from schizophrenia induced by genetic deficiency and various environmental stressors^2^. Dopaminergic hyperfunction and glutamatergic hypofunction have been linked to some aspects of schizophrenia^3^,^4^,^5^, and specific antipsychotic drugs targeting dopamine and glutamate systems have shown therapeutic effects on schizophrenic patients^6^. Nevertheless, the molecular mechanisms underlying the pathogenesis of schizophrenia are largely undefined.

*Disrupted-in-schizophrenia 1* (DISC1) has emerged as a convincing susceptibility factor for mental disorders including schizophrenia and depression^7^. Complex roles of DISC1 in neurons have been highlighted, in a view of its diverse cellular functions in various subcellular locations mediated by several interacting partners^8^. For instance, the centrosome-associated DISC1-NDEL1 complex regulates neuronal migration in collaboration with dynein^9^, while mitochondrion-associated DISC1 regulates mitochondrial calcium dynamics and transport of axonal mitochondria^10^,^11^. At the synapse, DISC1 modulates spine and synaptic maintenance via Rac1 signaling and TNIK^12^,^13^. Thus, DISC1 is a multifunctional protein involved in multiple signaling pathways in a context-dependent manner.

Endoplasmic reticulum (ER), a continuous membrane system distributed throughout the cell, plays critical roles in fundamental cellular functions. Especially, ER regulates intracellular calcium homeostasis and harbors various signaling components^14^. Fine regulation of neuronal processes encompassing axonal growth, synaptic plasticity, and neuronal excitability requires ER-mediated calcium buffering function^15^,^16^. ER calcium dynamics is elaborately controlled by several resident proteins, such as inositol-1,4,5-trisphosphate receptors (IP3Rs), ryanodine receptors (RyRs), and smooth ER calcium ATPase (SERCA)^17^. Under basal conditions or in response to environmental signals, ER-stored calcium is released through IP3Rs and RyRs or cytosolic calcium is reabsorbed back to ER via SERCA, followed by the activation of various intracellular signaling pathways regulated by calcium^17^.

The exocyst complex, composed of eight subunits, is conserved across species from yeast to mammals and shown to be involved in cellular deliveries, such as exocytosis or protein trafficking^18^,^19^. Possible association between ER and EXOC1, a component of the exocyst complex, was initially suggested based on the finding that EXOC1 regulates cortical ER inheritance and accumulation of Sec61p, a core complex involved in protein translocation across the ER membrane^20^. This hypothesis was supported by independent studies showing physical and functional interactions of subunits of the exocyst complex with the Sec61p complex^21^,^22^ and IP3Rs^23^.

Here, we propose that DISC1 functions in ER calcium dynamics in collaboration with EXOC1, thereby providing a potential molecular link between perturbed ER calcium regulation and pathogenesis of schizophrenia.

## Methods

### Antibodies

Rabbit anti-mitofilin (NB100-1919), rabbit anti-calnexin (SPA-865) and mouse anti-α-tubulin (DM1A) antibodies were purchased from NOVUS Biologicals, Assay Designs and Santa Cruz Biotechnology, respectively. Goat anti-mouse DISC1 (N16, SC-47990), goat and rabbit anti-IP3R1 (SC-6093, SC-28614) antibodies were purchased from Santa Cruz Biotechnology. Rabbit anti-EXOC1 antibody (11690-1-AP) was purchased from Proteintech. Rabbit anti-phospho IP3R (Ser1756, 3760S) was purchased from Cell Signaling. Mouse and rabbit anti-GFP (SC-9996, A11122) antibodies were acquired from Santa Cruz Biotechnology and Molecular Probes, respectively. Mouse and rabbit anti-Flag (F1804, SC-807) antibodies were purchased from Sigma-Aldrich and Santa Cruz Biotechnology, respectively.

### Plasmids and shRNA constructs

Flag-DISC1, Flag-mouse DISC1 (Flag-mDISC1) and Flag-DISC1 fragments were generated in pFlag-cmv2 vector (Sigma-Aldrich). GFP-DISC1, GFP-DISC1 fragments, GFP-EXOC1 and GFP-EXOC1 fragments were generated in pEGFP-C3 vector (Clontech). RFP-EXOC1 was generated in pmCherry-C1 vector (Clontech). GFP-ER made with *Arabidopsis* Bip ER signal sequence, retention signal and GFP was kindly provided by Inhwan Hwang (Department of Life Sciences, Pohang University of Science and Technology, Korea). shRNA constructs for DISC1 were generated by targeting two sequences (*shRNA1*: AAGAGTGCAGCAGCCCCTACT, nucleotide positions 301–321; *shRNA2*: AAGGAAAATACTATGAAGTAC, nucleotide positions 1993–2013). DISC1 shRNA2 was mainly used for human DISC1 knockdown in HEK293 cells. Mouse DISC1 (mDISC1) shRNA used in this study has been described previously^10^ (AAGGCAAACACTGTGAAGTGC, nucleotide positions 1984–2004). EXOC1 shRNA target sequences were selected according to the report^24^ (CCCGACTATATGAAAGAGAAA, nucleotide positions 1244–1264 for human *exoc1* and 1265–1285 for mouse *exoc1* genes).

### Mouse lines

Pregnant CrljBgi:CD-1 (Institute for Cancer Research [ICR]) and B57BL/6 mice were purchased from Hyochang Science and embryos were processed for the culture of primary neurons. Genetically engineered DISC1 mutant (*Δ*2–3) mice were provided by Dr. Kaibuchi^25^. All animal procedures were approved by the Pohang University of Science and Technology Institutional Animal Care and Use Committee. All experiments were carried out in accordance with the approved guidelines.

### Cell culture and transfection

The HEK293 cell line was maintained in DMEM (Hyclone) supplemented with 10% (v/v) fetal bovine serum (Thermo Scientific) and penicillin/streptomycin (GIBCO). Mouse embryonic hippocampal neurons were maintained in Neurobasal medium (GIBCO) supplemented with B27, glutamine and penicillin/streptomycin. Lipofectamine 2000 (Invitrogen) was used for transfection of HEK293 cells. Briefly, DNA constructs were mixed with Lipofectamine 2000 in Opti-MEM (Invitrogen), and the mixtures added to cells after incubation for 20 min. The calcium phosphate method was used for transfection of cultured embryonic hippocampal neurons as described previously^26^. DNA constructs were mixed with 2.5 M calcium chloride in 2X HEPES-buffered saline and the mixtures added to neurons after incubation for 20 min. After 1 h, neurons were washed in 1X Hank's balanced salt solution (GIBCO) three times and incubated at 37°C and 5% CO2.

### Subcellular fractionation

Subcellular fractionation was carried out as described previously^27^. Briefly, cultured cells were harvested in phosphate-buffered saline (PBS) and centrifuged for 5 min at 1,400×g, 4°C. The cell pellet was suspended in MTE buffer (270 mM D-mannitol, 10 mM Tris-Cl, pH7.4, 0.1 mM EDTA, protease inhibitor cocktail [Roche]) and disrupted via dounce homogenization. The homogenate was centrifuged for 10 min at 2,500×g, 4°C. The supernatant was collected and centrifuged for 10 min at 15,000×g, 4°C. The pellet was collected as the mitochondrial fraction, while the supernatant was loaded on a sucrose gradient and centrifuged for 70 min at 152,000×g, 4°C. The top portion was collected as the cytosolic fraction and the white band was collected with syringe and centrifuged for an additional 45 min at 126,000×g, 4°C. The pellet was collected as the ER fraction. The protein concentration was determined with the Bradford assay (Bio-Rad Laboratories) and the equivalent quantities of protein subjected to SDS-PAGE, followed by Western blotting.

### Immunocytochemistry

Transfected HEK293 cells or cultured hippocampal neurons of ICR mouse embryos at days 10 *in vitro* (DIV10) were transfected on 18 mm coverslips and washed once in PBS. Cells were fixed in 4% paraformaldehyde for 20 min at room temperature and permeabilized in 0.25% Triton X-100, followed by blocking in 10% normal goat serum for 30 min at room temperature. Cells were incubated with antibodies (diluted 1:200–500) for 2 h at 37°C. After three washes, cells were treated with Alexa Fluor 488-, 568-, and 647-conjugated antibodies (Molecular Probes) for 1 h at 37°C. All images were obtained using a laser scanning confocal microscope (model FV1000; OLYMPUS) using an UPLSAPO 60×/1.35 NA oil objective and FV10-ASW2.0 fluoviewer software was used for image analysis.

### Immunoprecipitation

Cultured HEK293 cells were sonicated for lysis in Nonidet P-40 lysis buffer (50 mM Tris, pH 8.0, 150 mM NaCl, 1% Nonidet P-40, 5 mM EDTA, 1X EDTA-free protease inhibitor cocktail). For immunoprecipitation of IP3R1 with goat anti-IP3R1 antibodies, 0.5% Triton-X 100 was added to Nonidet P-40 buffer. Cell lysates were centrifuged for 10 min at 12,000×g and the supernatants were incubated with 1–2 μg antibodies for 10–15 h at 4°C with constant rotation. Protein-A agarose beads (Roche) washed in Nonidet P-40 buffer three times were added, followed by incubation for 3–5 h at 4°C with constant rotation. Beads were collected via centrifugation, washed three times, and suspended in 2X SDS sampling buffer.

### Immunogold electron microscopy

Immunogold electron microscopy was carried out as described previously^10^. Briefly, samples were fixed in 2.5% glutaraldehyde in 0.1 M sodium cacodylate buffer (pH 7.0, Sigma-Aldrich) for 30 min at 4°C. Fixed HEK293 cells expressing Flag-DISC1 were sliced using an ultramicrotome (RMC MTXL), mounted on nickel grids, etched with 10% hydrogen peroxide for 30 min, and incubated with rabbit anti-Flag antibodies (1:100) at 4°C overnight. After washing three times, sections were incubated with 10 nm gold particle-conjugated anti-rabbit IgG (1:20, Sigma-Aldrich) for 1 h. For immunogold double-labeling, HEK293 cell sections transfected with Flag-DISC1 and adult brain sections from ICR adult mouse were obtained in nickel and gold grids, respectively. Sections of HEK293 cells were incubated with mouse anti-Flag and rabbit anti-calnexin antibodies at 4°C overnight, followed by incubation with 6 nm gold particle-conjugated anti-mouse IgG (1:20, Sigma-Aldrich) and 15 nm gold particle-conjugated anti-rabbit IgG (1:20, Sigma-Aldrich) for 1 h. Adult mouse brain sections were incubated with goat anti-mDISC1 and rabbit anti-calnexin antibodies at 4°C overnight, followed by incubation with 6 nm gold particle-conjugated anti-goat IgG (1:20, Sigma-Aldrich) and 15 nm gold particle-conjugated anti-rabbit IgG (1:20) for 1 h. Labeled and stained sections were viewed at 120 kV under an electron microscope (model JEM1011; JEOL).

### Trypsin sensitivity assay

Trypsin sensitivity analysis of ER proteins was carried out as described previously^10^,^28^. ER fractions from transfected HEK293 cells were equally divided into eight aliquots. Four of the aliquots were permeabilized for 5 min with 2% Triton X-100 treatment and the other four left untreated. Aliquots were treated with increasing doses of trypsin and incubated for 30 min on ice. After trypsin treatment, aliquots were resuspended in 2X SDS sampling buffer and subjected to SDS-PAGE, followed by Western blotting. Band intensities were analyzed with the Image J software (National Institutes of Health).

### Live calcium imaging

Calcium imaging was carried out as described previously^29^,^30^. Differentiated mouse Cath.-a-differentiated (CAD) cells or cultured hippocampal neurons from ICR mouse embryos at DIV2 were transfected with shRNA constructs and cultured for additional 5 days prior to calcium imaging. Calcium imaging in hippocampal neurons cultured from wild-type and DISC1 mutant (*Δ*2–3) mouse embryos was carried out at DIV7. To assess the effects of antipsychotics and DRD1 antagonist, cultured hippocampal neurons at DIV5 were treated with 0.1 μM haloperidol (Sigma-Aldrich), 1 μM clozapine (Sigma-Aldrich) or 10 μM SCH23390 (Sigma-Aldrich), prior to calcium imaging at DIV7. To assess the effects of dopamine, 10 μM dopamine (Sigma-Aldrich) were simultaneously treated with ATP (Sigma-Aldrich) in hippocampal neurons at DIV7. HEK293 cells transfected with shRNA constructs were diluted and replated in new culture dishes two days after transfection. Cells were cultured for further three days, followed by calcium imaging. The intracellular calcium level was measured using Fura2/AM (Invitrogen). HEK293 cells were loaded with 10 μM Fura-2/AM in calcium-free Krebs-Ringer buffer (5.5 mM HEPES, pH7.4, 140 mM NaCl, 5 mM KCl, 0.5 mM MgCl2, 10 mM glucose, 100 μM EGTA). Differentiated CAD cells and primary cultured neurons were loaded with 10 μM Fura-2/AM in Krebs-Ringer calcium buffer (10 mM HEPES, pH7.4, 126 mM NaCl, 4 mM KCl, 2 mM CaCl2, 1 mM MgCl2, and 4.2 mM glucose). After incubation for 30 min at 37°C, cells were transferred to the chamber and treated with ATP, bradykinin (Sigma-Aldrich) or caffeine (Sigma-Aldrich) to induce ER calcium release. The fluorescence intensities were recorded in MetaFluor or Metamorph software at an interval of 1 s using an inverted microscope (model IX71; OLYMPUS) with an UPLSAPO 20×/0.75 NA objective and a CCD camera (model C9100-13; Hamamatsu Photonics). To measure ER calcium, CAD cells were transfected with shRNA constructs and ER-targeted aequorin (ER-AEQ), as described previously^31^. The cells were cultured for three days and replated in new 24-well culture dishes. ER-stored calcium was depleted by 10 μM *tert*-butylhydroquinone (TBHQ, Sigma-Aldrich) in calcium-free Krebs-Ringer buffer for 20 min at room temperature. Cells were washed, and incubated with 5 μM coelenterazine (Cayman Chemical) in EGTA- and calcium-free buffer for 1 h at room temperature to reconstitute ER-AEQ and exposed with 10 mM CaCl2. ER-AEQ luminescence intensities were recorded by a plate reader (Tecan, infinite M200 pro).

## Results

### DISC1 localizes to the endoplasmic reticulum (ER)

**Figure 1:**
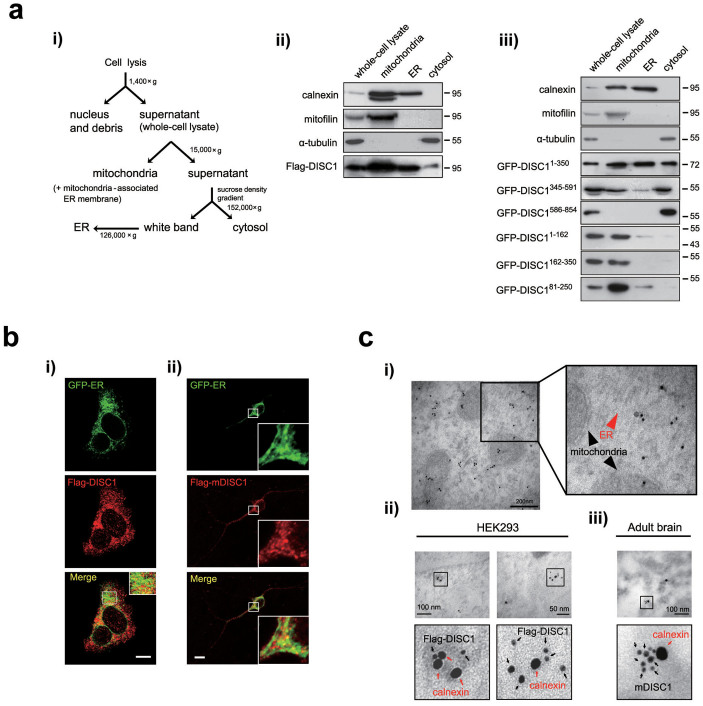
Localization of DISC1 in the ER. (a) The subcellular fractionation procedure using sucrose gradients (*1*) and subcellular localization of DISC1 (*2i*) or DISC1 fragments (*3i*) in HEK293 cells. Calnexin, mitofilin and α-tubulin were used as ER, mitochondrial and cytosolic markers, respectively. (b) Co-localization between Flag-DISC1 and GFP-ER in HEK293 cells (*i*), Flag-mDISC1 and GFP-ER in mouse hippocampal neurons at DIV10 (*ii*). Scale bars represent 10 μm. (c) Electron micrograph with pre-embedding immunogold staining. Ultrastructure of Flag-DISC1 in HEK293 cells showing the existence of DISC1 in the ER (red arrowhead) and mitochondria (black arrowheads) (*i*). Double immunogold labeling of Flag-DISC1 with calnexin in HEK293 cells (*ii*) and endogenous mDISC1 with calnexin in adult mouse brain sections (*iii*). Immunogold particles (6 nm) for Flag-DISC1 and endogenous mDISC1 are indicated with black arrows and 15 nm immunogold particles for calnexin with red arrows. The magnified images are represented with bold lines and scale bars represent 200 nm (*i*) and 100 nm or 50 nm (*ii*, *iii*). Western blotting was performed under the same experimental conditions in each panel and full-length blots are presented in Supplementary Fig. 9.

DISC1 has been shown to function in multiple cellular compartments^8^. To further clarify the diverse roles of DISC1, we performed a series of subcellular fractionation experiment using sucrose density gradients in HEK293 cells transfected with the Flag-DISC1 (Fig. 1a i). Calnexin, mitofilin, and α-tubulin were used as ER, mitochondrial, and cytosolic markers, respectively. In addition to the ER fraction, calnexin was detected in mitochondrial fraction which reflects its expression in the mitochondria-associated ER membrane fraction^27^,^32^. Localization of DISC1 to mitochondria was observed, which is consistent with a previous report^10^, and a significant localization of DISC1 to the ER fraction was also evident (Fig. 1a ii). To determine the specific regions of DISC1 protein critical for ER localization, HEK293 cells were transfected with DISC1 fragments and subjected to subcellular fractionation. DISC1^1–350^ was predominantly detected in the ER fraction, while DISC1^345–591^ and DISC1^586–854^ were either significantly reduced or not detected in the ER (Fig. 1a iii). Upon subdivision of DISC1^1–350^ into three fragments, ER localization was not significantly detected (Fig. 1a iii). The data indicate that ER localization of DISC1 is mainly governed by residues 1–350. We also analyzed ER localization of DISC1 using immunocytochemistry. We used a fusion construct as an ER luminal marker (GFP-ER) as described previously^33^. DISC1 and mouse DISC1 (mDISC1) displayed partial, but significant co-localization with GFP-ER in HEK293 cells (Fig. 1b i) and in cultured hippocampal neurons at DIV10 (Fig. 1b ii) while DISC1 deletion mutant lacking residues 1–350 (DISC1*^Δ^*^1–350^) did not show clear ER localization (Supplementary Fig. 1a). To further confirm the ER localization of DISC1, immunogold electron microscopy (IGEM) was employed. DISC1 was detected in the ER-like membranous structure of HEK293 cells (Fig. 1c i). In double IGEM analysis, Flag-DISC1 was detected in proximity to calnexin in transfected HEK293 cells (Fig. 1c ii). Consistently, endogenous mDISC1 detected by goat anti-mDISC1 antibody was localized to the proximity of calnexin in adult mouse brain sections (Fig. 1c iii). These data indicate that DISC1 localizes to ER, implying functional links between DISC1 and ER.

### DISC1 interacts with EXOC1

**Figure 2:**
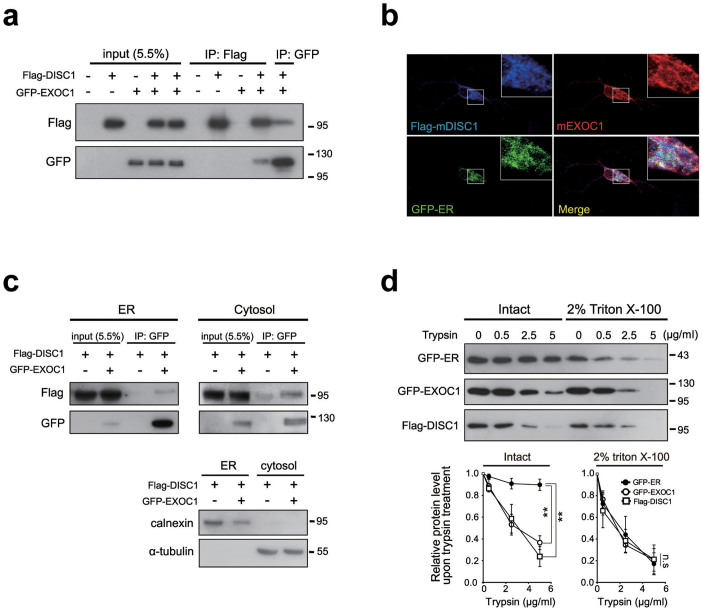
Interaction between DISC1 and EXOC1. (a) Co-immunoprecipitation of Flag-DISC1 with GFP-EXOC1 in HEK293 cells. (b) Co-localization of Flag-mDISC1 and endogenous mEXOC1 in the ER of mouse hippocampal neurons at DIV10. Scale bars represent 10 μm. (c) Co-immunoprecipitation of Flag-DISC1 with GFP-EXOC1 in ER and cytosolic fractions of HEK293 cells. 400 μg of ER and 1 mg cytosolic protein preparations were used. (d) Trypsin sensitivity of Flag-DISC1 and GFP-EXOC1 in the ER fraction of HEK293 cells. GFP-ER (ER luminal construct), Flag-DISC1 and GFP-EXOC1 in intact or permeabilized aliquots of the ER fraction were degraded by trypsin in a dose-dependent manner. Band intensities were quantified with Image J and normalized to the control lane (no trypsin treatment) (n = 3). Error bars represent mean ± SEM. **; *P* < 0.01 (two-way ANOVA). Western blotting was performed under the same experimental conditions in each panel and full-length blots are presented in Supplementary Fig 9.

Localization of DISC1 to various cellular compartments is generally mediated by specific binding partners^8^. We tested a number of potential binding partners of DISC1 in the ER, based on the available DISC1 interactomes^34^. Following examination of candidate proteins via co-immunoprecipitation in heterologous systems, EXOC1 was identified as a potential binding partner of DISC1. EXOC1 is a component of the exocyst complex and earlier reports suggest the enrichment of some exocyst components in ER^20^,^23^. In our results, DISC1 was co-immunoprecipitated with EXOC1 (Fig. 2a). Consistently, we observed strong overlap of signals for Flag-mDISC1, endogenous mouse EXOC1 (mEXOC1) and GFP-ER in hippocampal neurons (Fig. 2b). However, EXOC1 did not show strong co-localization with DISC1*^Δ^*^1–350^, indicating that amino acid residues 1–350 of DISC1 is responsible for both ER localization and interaction with EXOC1 (supplementary Fig. 1b). In an attempt to define the domain of EXOC1 responsible for interaction with DISC1 using co-immunoprecipitation with various fragments of EXOC1, we found that the interaction interface appears complex rather broadly dispersed in the EXOC1 protein (Supplementary Fig. 2). To clarify the subcellular compartment housing the interaction, we performed subcellular fractionation, followed by co-immunoprecipitation, in HEK293 cells. DISC1 effectively interacted with EXOC1 in ER and cytosolic fractions, suggesting association between DISC1 and EXOC1 in both the ER and cytosol (Fig. 2c). To determine the nature of DISC1 association with ER, we performed the trypsin sensitivity assay. GFP-ER, an ER luminal marker, was degraded by trypsin in a dose-dependent manner only upon treatment with Triton X-100. However, DISC1 and EXOC1 were efficiently degraded by trypsin, regardless of Triton X-100 treatment, indicating that DISC1 is associated with EXOC1 on the ER surface (Fig. 2d). Based on these findings, we propose that EXOC1 is an interacting partner of DISC1 in the ER.

### DISC1 is associated with IP3R1 in cooperation with EXOC1

**Figure 3:**
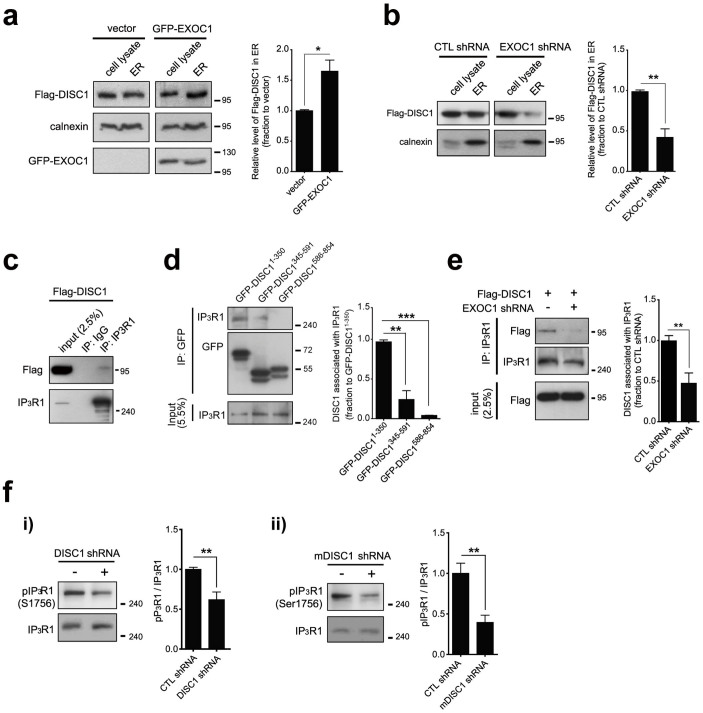
Association with IP3R1 of DISC1 in cooperation with EXOC1. (a) Enhanced ER localization of Flag-DISC1 upon EXOC1 overexpression in HEK293 cells. Statistical quantification of ER localization of Flag-DISC1 was performed with densitometric analysis using Image J. DISC1 level was normalized to calnexin (n = 3). (b) Reduced ER localization of Flag-DISC1 upon EXOC1 knockdown in HEK293 cells. Statistical quantification of ER localization of Flag-DISC1 was performed with densitometric analysis using Image J. DISC1 level was normalized to calnexin (n = 3). (c) Co-immunoprecipitation of Flag-DISC1 with endogenous IP3R1 in HEK293 cells. (d) Co-immunoprecipitation of three DISC1 fragments (residues 1–350, 345–591, 586–854) with endogenous IP3R1. Statistical quantification of the IP3R1 binding levels of DISC1 was carried out via densitometric analysis using Image J (n = 3). (e) Reduced IP3R1 interaction with DISC1 upon EXOC1 knockdown in HEK293 cells. Statistical quantification of the IP3R1 binding levels of DISC1 was carried out via densitometric analysis using Image J. DISC1 level in IP lane was normalized to input lane (n = 6). (f) Reduced PKA-mediated phosphorylation of IP3R1 in DISC1 knockdown HEK293 cells (*i*) and mouse neuroblastoma CAD cells (*ii*) (n = 6 for *i*, 7 for *ii*). Error bars represent means ± SEM. *; *P* < 0.05, **; *P* < 0.01, ***; *P* < 0.001 (two-tailed *t*-test). Western blotting was performed under the same experimental conditions in each panel and full-length blots are presented in Supplementary Fig 9.

Using the shRNA constructs generated against DISC1 and EXOC1^10^,^24^ (Supplementary Fig. 3), we examined potential contribution of EXOC1 in ER localization of DISC1. Remarkably, overexpression of EXOC1 led to an increase of DISC1 level in the ER fraction (Fig. 3a). On the other hand, the DISC1 level in the ER fraction was significantly decreased upon EXOC1 knockdown (Fig. 3b). Interestingly, the exocyst complex was proposed to be involved in IP3-mediated calcium signaling, in view of the interaction with IP3R1 in the ER^23^. Accordingly, we further examined whether DISC1 interacts with IP3R1 and we found that DISC1 is co-immunoprecipitated with endogenous IP3R1 in HEK293 cells (Fig. 3c). Moreover, 1–350 residues of DISC1 contributed significantly to interaction with IP3R1, implying that the ER localization of DISC1 is linked to IP3R1 interaction (Fig. 3d). Notably, EXOC1 knockdown caused a significant decrease in interaction between DISC1 and IP3R1 (Fig. 3e). Although complete understanding of the roles for EXOC1 in DISC1 action in ER requires further analysis, our findings suggest that EXOC1is involved in function of DISC1 in ER.

It is relatively difficult to directly analyze the mechanistic details of the interaction due to the structural complexity and big size of IP3R1 protein^35^. However, the regulation of IP3R1 activity by phosphorylation is relatively well established, and cAMP-dependent protein kinase A (PKA) that is functionally linked with DISC1^36^ is proposed to be a key regulator^37^. Therefore, we examined the possibility of PKA phosphorylation regulated by DISC1 as a potential mechanistic route to alter IP3R1 activity. We assessed PKA-mediated phosphorylation of IP3R1 using an antibody against phosphorylated serine 1756 residue of IP3R1 that is critical for IP3R1 activities^38^. IP3R1 activities were significantly decreased in DISC1 knockdown cells (Fig. 3f i) and mouse neuroblastoma CAD (Cath.-a-differentiated) cells (Fig. 3f ii) by specific shRNAs. On the other hand, decrease of PKA-mediated phosphorylation of IP3R1 was marginal in EXOC1 knockdown cells compared to DISC1 knockdown cells, suggesting that EXOC1's function on IP3R1 is rather indirect in contrast to DISC1 (Supplementary Fig. 4). Also, we observed that the increased PKA-mediated phosphorylation of IP3R1 by forskolin, an activator of adenylyl cyclase, leads to a decrease in ER calcium release, supporting the reports that PKA-mediated phosphorylation of IP3Rs regulates the IP3-mediated ER calcium dynamics (Supplementary Fig. 5). These results indicate that the regulation of IP3R1 by DISC1 involves PKA phosphorylation.

### DISC1 and EXOC1 regulate ER calcium dynamics

**Figure 4:**
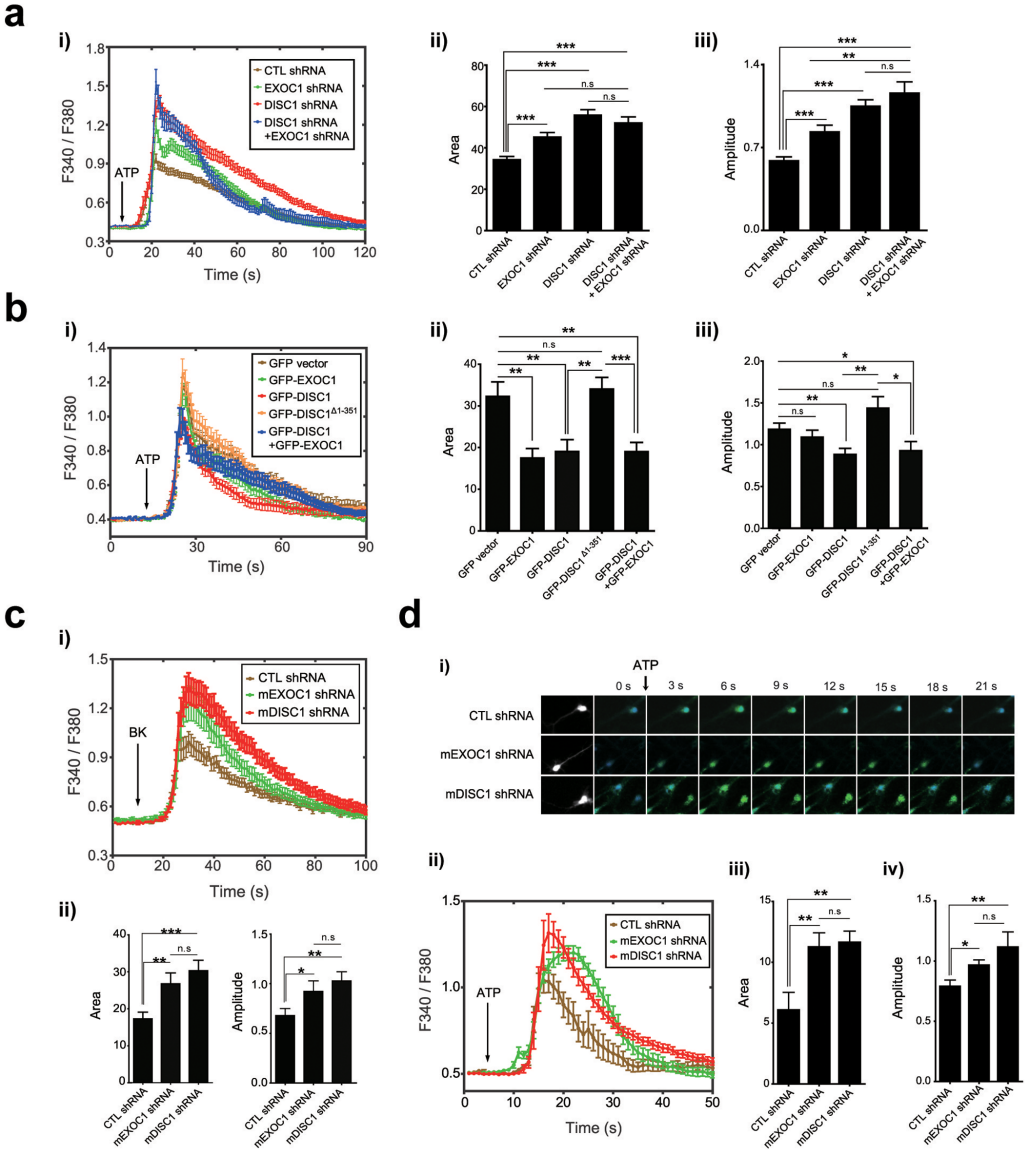
Regulation of ER calcium dynamics by DISC1 and EXOC1. (a) Enhanced IP3R-dependent ER calcium dynamics upon DISC1 or EXOC1 knockdown in HEK293 cells. Calcium response graph under 50 μM ATP stimulation (*i*), statistically analyzed average area (*ii*) and amplitudes (*iii*) of the graph (n = 30 for control shRNA, 29 for EXOC1 shRNA, 42 for DISC1 shRNA, 21 for DISC1 shRNA + EXOC1 shRNA). (b) Reduced IP3R-dependent ER calcium dynamics in HEK293 cells overexpressing DISC1 or EXOC1 under 50 μM ATP stimulation. Based on ER calcium response curves (*i*), statistically analyzed average area (*ii*) and amplitude (*iii*) were shown (n = 24 for GFP vector, 21 for GFP-EXOC1, 17 for GFP-DISC1, 35 for GFP-DISC1 *Δ*1-351, 18 for GFP-DISC1+GFP-EXOC1). (c) ER calcium response curves under 50 nM bradykinin stimulation in mDISC1 and mEXOC1 knockdown neuroblastoma CAD (Cath.-a-differentiated) cells (*i*). Area and amplitude of ER calcium response curve under 50 nM BK (bradykinin) were analyzed (*ii*) (n = 41 for control shRNA, 26 for mEXOC1 shRNA, 39 for mDISC1 shRNA). (d) Enhanced IP3R-dependent ER calcium dynamics upon mDISC1 or mEXOC1 knockdown in DIV7 mouse hippocampal neurons. Representative live images of calcium response to ATP stimulation in control, mEXOC1, and mDISC1 knockdown conditions (*i*), calcium response graph under 10 mM ATP stimulation (*ii*), statistically analyzed average area (*iii*) and amplitudes (*iv*) of the graph (n = 17 for control shRNA, 13 for mEXOC1 shRNA, 11 for mDISC1 shRNA). Error bars represent means ± SEM. *; *P* < 0.05, **; *P* < 0.01, ***; *P* < 0.001 (two-tailed *t*-test).

As neuronal IP3Rs play critical roles in ER calcium dynamics^15^, we investigated whether DISC1 and EXOC1 affect IP3-mediated ER calcium dynamics. We initially examined whether DISC1 and EXOC1 loss-of-function affect ER calcium dynamics in heterologous systems using ATP, an agonist for IP3R-evoked ER calcium release^39^. The area and amplitude of ATP-induced calcium response curves were remarkably increased in DISC1 and EXOC1 knockdown cells (Fig. 4a i–iii). Conversely, overexpression of DISC1 or EXOC1 reduced ATP-induced ER calcium release in HEK293 cells (Fig. 4b i–iii). Knockdown or overexpression of both DISC1 and EXOC1 did not exert a significant synergistic effect, implying that they regulate ER calcium dynamics through overlapping pathway. While DISC1 overexpression reversed the increased ER calcium release in EXOC1 knockdown cells, EXOC1 overexpression could not effectively rescue abnormal increased ER calcium release in DISC1 knockdown cells (Supplementary Fig. 6a–c). These results support the idea that DISC1 and EXOC1 function in an overlapping pathway to regulate the ER calcium dynamics and suggest that DISC1 functions downstream of EXOC1. In addition, overexpression of a DISC1*^Δ^*^1–350^ did not reduce the ER calcium response, indicating that ER localization and interaction with IP3R1 are important for DISC1 to regulate ER calcium dynamics (Fig. 4b ii, iii).

Because DISC1 localizes to mitochondria and plays essential role in mitochondrial calcium dynamics^10^, we examined whether abnormal ER calcium dynamics in DISC1 or EXOC1-deficient conditions is derived from perturbation of mitochondrial calcium uptake. To address this point, live calcium imaging was performed in same experimental setting except for the ruthenium red treatment to suppress mitochondrial calcium uptake^40^. The effects of DISC1 and EXOC1 knockdown on ER calcium dynamics were still maintained with ruthenium red, indicating that contribution of a perturbed mitochondrial calcium homeostasis to the abnormalities in ER calcium dynamics caused by DISC1-deficiency is marginal, if there is (Supplementary Fig. 7). In addition, basal ER calcium levels were not significantly changed in DISC1- and EXOC1-knockdown cells, showing that the abnormal increases of IP3R-mediated ER calcium dynamics are not likely to be caused by an altered basal ER calcium levels (Supplementary Fig. 8a). Also, RyRs-mediated ER calcium release stimulated by caffeine^41^ was not affected by DISC1 and EXOC1 knockdown (Supplementary Fig. 8b), further supporting the notion that DISC1 and EXOC1 regulate ER calcium dynamics via IP3Rs.

Similar results were observed in response to bradykinin, another stimulant that induces IP3-mediated ER calcium release in EXOC1- or DISC1-knockdown differentiated neuroblastoma CAD cells (Fig. 4c i, ii). Furthermore, mDISC1 or mEXOC1 knockdown enhanced ATP-induced ER calcium release in hippocampal neurons (Fig. 4d i, ii). The area and amplitude of calcium response curves in mDISC1 or mEXOC1 knockdown neurons were significantly greater than those obtained from the respective control groups (Fig. 4d iii, iv).

### Antipsychotic drugs reverse abnormal ER calcium dynamics in DISC1-deficient neurons

**Figure 5:**
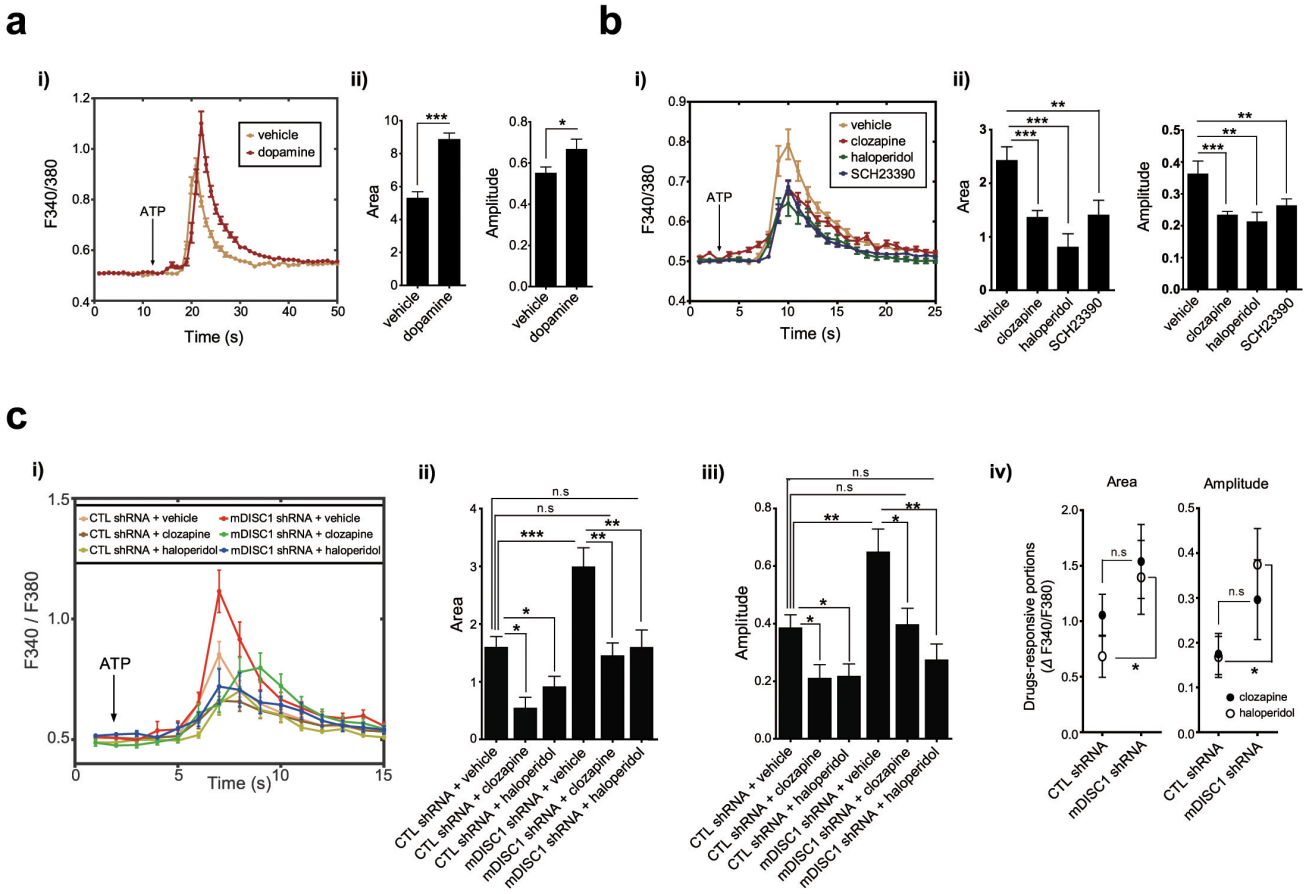
Reversed abnormal ER calcium dynamics with antipsychotic treatment in mDISC1 knockdown hippocampal neurons. (a) Increased calcium response upon dopamine treatment. ER calcium response curves to 10 mM ATP in hippocampal neurons treated with 10 μM dopamine (*i*). Average area and amplitude of response curve were analyzed (*ii*) (n = 66 for vehicle, 59 for dopamine). (b) Decreased calcium response to ATP upon treatment with antipsychotic drugs (clozapine and haloperidol) or a specific antagonist for dopamine D1 receptor, SCH23390. ER calcium response curves to ATP (30 mM) in hippocampal neurons treated with clozapine (1 μM), haloperidol (0.1 μM) or SCH23390 (10 μM) (*i*). Average area and amplitude of response curve were analyzed (*ii*) (n = 42 for vehicle, 59 for clozapine, 37 for haloperidol and 50 for SCH23390). (c) Reversal of the exaggerated ER calcium dynamics caused upon mDISC1 knockdown in hippocampal neurons treated with 1 μM clozapine or 0.1 μM haloperidol. Live ER calcium response graph under 30 mM ATP stimulation (*i*), statistically analyzed average area (*ii*), amplitudes (*iii*) and drug-responsive portions (*iv*) of the graph (n = 31 for control shRNA+vehicle, 11 for control shRNA+clozapine, 22 for control shRNA+haloperidol, 29 for mDISC1 shRNA+vehicle, 14 for mDISC1 shRNA+clozapine, 17 for mDISC1 shRNA+haloperidol). Error bars represent means ± SEM, *; *P* < 0.05, **; *P* < 0.01, ***; *P* < 0.001 (two-tailed *t*-test).

Dopamine D1 receptor (DRD1) and D2 receptor (DRD2) regulate adenylyl cyclase and cyclic AMP signaling through Gs/olf and Gi/o proteins, respectively^42^. On the other hand, DRD1-DRD2 heteromers can couple to Gq protein in the brain and their activation leads to rapid ER calcium release via Gq-phospholipase C-IP3 pathway^43^,^44^. Consistent with these reports, we observed an increase in ER calcium release in hippocampal neurons upon dopamine treatment (Fig. 5a i, ii) and a decrease in ER calcium release upon SCH23390 treatment, a DRD1 antagonist (Fig. 5b i, ii). Also, it has been shown that administration of antipsychotic drugs, which mainly target DRD2, inhibit IP3-dependent calcium release^45^, indicating that dopamine receptor-mediated signaling is tightly associated with ER calcium dynamics. Therefore, we further examined whether ER calcium dynamics under control of DISC1 is affected by antipsychotics. Cultured hippocampal neurons were treated with clozapine or haloperidol for 2 days before calcium imaging and stimulated with ATP (Fig. 5c i). Upon treatment of mDISC1 knockdown hippocampal neurons with the drugs, exaggerated calcium responses were effectively reversed (Fig. 5c ii, iii). Also, mDISC1 knockdown neurons showed larger responsiveness to drugs than control neurons, indicated by increased drug-responsive portions of calcium response curves (Fig. 5c iv). These results suggest that perturbed ER calcium dynamics caused by DISC1 deficiency is linked to disruption of the neuronal environment associated with schizophrenia.

### Neurons from DISC1-deficient mouse show abnormal ER calcium dynamics

**Figure 6:**
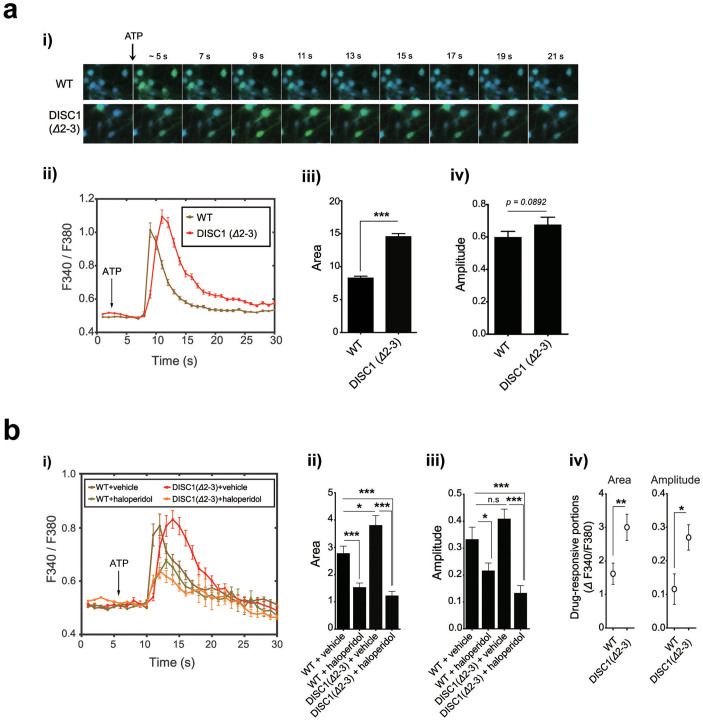
Abnormal ER calcium dynamics of hippocampal neurons from DISC1 (*Δ*2-3) mice. (a) Live calcium images of cultured hippocampal neurons of wild-type (WT) and DISC1 (*Δ*2-3) mouse embryos in response to ATP (*i*). ER calcium response graph under 10 mM ATP (*ii*), statistically analyzed area (*iii*), amplitude (*iv*) (n = 88 for WT, 74 for DISC1 (*Δ*2-3)). (b) Reversal of the exaggerated ER calcium dynamics in DISC1 (*Δ*2-3) hippocampal neurons by haloperidol (0.1 μM). From the ER calcium response curves upon 30 mM ATP stimulation (*i*), average area under curves (*ii*), amplitudes (*iii*) and drug-responsive portions (*iv*) of the curves were analyzed. (n = 31 for WT+vehicle, 28 for WT+haloperidol, 26 for DISC1 (*Δ*2-3)+vehicle, 27 for DISC1 (*Δ*2-3)+haloperidol). Error bars represent means ± SEM. *; *P* < 0.05, **; *P* < 0.01, ***; *P* < 0.001 (two-tailed *t*-test).

To verify the roles of DISC1 in the regulation of ER calcium dynamics in more physiologically relevant setting, we employed DISC1 mutant mouse lacking exons 2 and 3 (*Δ*2–3) generated by homologous recombination^25^. In hippocampal neurons of DISC1 (*Δ*2–3), area under the curves of calcium response induced by ATP was larger than that in wild-type (WT) hippocampal neurons, similar to DISC1 knockdown (Fig. 6a i–iii). The increase in amplitude of calcium response curves of DISC1 (*Δ*2–3) neurons was less prominent compared to DISC1 knockdown cells, showing a tendency toward statistical significance (p = 0.0892, two-tailed *t-*test, Fig. 6a iv). The exaggerated calcium responses in DISC1 (*Δ*2–3) neurons were effectively reversed by haloperidol treatment (Fig. 6b i–iii) with greater responsiveness than in control neurons, similar to DISC1 knockdown (Fig. 6b iv). These results collectively show that the perturbation of the ER calcium response is associated with DISC1 loss-of-function in DISC1 mutant mouse, further supporting a link between DISC1 and ER calcium dynamics.

## Discussion

In this study, we propose that DISC1 has a novel intracellular function linked to regulation of the ER calcium dynamics via IP3Rs. DISC1 localizes to the ER outer surface likely assisted by EXOC1, a component of exocyst complex. Notably, the domain of DISC1 responsible for localization to the ER is also critical for IP3R1 interaction; this indicates that IP3R1 is the destination for DISC1 on ER. Although further analyses to reveal the roles for EXOC1 or exocyst complex in the recruitment and action of DISC1 on IP3R1 in the mechanistic point of view are needed in detail, our results are in the similar line to the previous reports that exocyst complex is involved in the recruitments of multiple cytosolic factors to specific subcellular regions, thereby regulating various cellular events^46^,^47^. Moreover, the exocyst complex on the ER has been shown to modulate intracellular calcium signaling and indeed physically interacts with IP3R^23^. Therefore, our results suggest a possible link between the exocyst complex and IP3R. Therefore, the EXOC1-mediated recruitment of DISC1 to IP3R may act as a mechanism linking the exocyst complex and IP3R. Our results also indicate that once recruited to IP3R complex, DISC1 has impact on IP3R1 activity by affecting PKA-mediated phosphorylation. Multiple previous reports showed that the phosphorylation of IP3R1 by PKA leads to inhibition of calcium release from ER in various cellular contexts^48^,^49^,^50^,^51^. Therefore, it is plausible that DISC1 deficiency causes deregulation of PKA-mediated phosphorylation of IP3R1, thereby inducing abnormal ER calcium dynamics.

Given that DISC1 is considered to be a major schizophrenia susceptibility factor, the link between DISC1 and ER calcium dynamics shown in this study hints at a connection between schizophrenia pathogenesis and the fundamental cellular processes associated with intracellular calcium homeostasis. Indeed, multiple studies have proposed that intracellular calcium is important to various mental disorders, including schizophrenia. For example, Jimerson *et al*. reported that the calcium levels in the cerebrospinal fluid of schizophrenia patients are correlated with the remission of positive symptoms^52^. The expression patterns of proteins involved in the intracellular calcium network are significantly altered in the post-mortem brains of schizophrenia patients^53^,^54^. Ripova and co-workers reported that the basal levels of intracellular calcium are increased in the platelets of schizophrenic patients^55^. Significant elevations of ER calcium release are detected in the platelets of schizophrenia patients upon receptor stimulation with thrombin or glutamate^56^,^57^. More recently, phospholipase C-β1 knockout mice were shown to exhibit schizophrenia-associated behavioral endophenotypes^58^. These reports consistently suggest that abnormalities in intracellular calcium homeostasis, including ER calcium dynamics, are associated with the pathogenesis of schizophrenia. However, the underlying molecular mechanisms were previously unknown. Here, we show that DISC1 directly participates in regulating ER calcium dynamics. This finding may mechanistically explain the interesting association between perturbed intracellular calcium dynamics and schizophrenia.

Notably, the exaggerated ER calcium response of DISC1-deficient neurons was found to be effectively reversed by antipsychotic drugs, suggesting the clinical relevance of our findings. Recent research has revealed an interesting connection between dopamine signaling and intracellular calcium homeostasis. Activation of dopamine receptors stimulates IP3 production, resulting in the release of calcium from the ER^44^,^59^, and increased release of calcium from the ER facilitates the release of somatodendritic dopamine^60^. Together, these reports indicate that dopamine signaling and ER calcium dynamics are positively interconnected. Thus, the exaggerated ER calcium response induced by a functional deficiency of DISC1 would result in hyperactive dopamine function, which is a key feature associated with positive symptoms of schizophrenia^3^. Perturbation of ER calcium dynamics is also connected to a hypoactive glutamate system, which is another core aspect of schizophrenia. Krupp *et al*. reported that increased cytosolic calcium inhibits the influx of calcium through NMDA receptors^61^. Thus, it is tempting to speculate that our findings may provide a platform for integrating the dopamine and glutamate hypotheses of schizophrenia at the level of intracellular signaling.

## Author Contributions

S.J.P. conceived the study, performed experiments, analyzed and interpreted data, and wrote the manuscript; J.J., N.L. performed experiments; S.M.K., Y.U.P., K.S.P. and H.L., K.K. (Kuroda), K.K. (Kaibuchi) provided vital reagents and experimental tools; M.D.N. design the experiments and interpreted data; S.K.P. conceived the study, analyzed and interpreted data, and wrote the manuscript.

## Supplementary Material

Supplementary Information Supplementary information
