# Supplementary material for: Disrupted-in-schizophrenia-1 (DISC1) Regulates Endoplasmic Reticulum Calcium Dynamics
Source: Sci Rep. 2015 Mar 3;5:8694. doi: 10.1038/srep08694 (PMC4346799; doi:10.1038/srep08694)

Disrupted-in-schizophrenia-1 (DISC1) Regulates Endoplasmic Reticulum Calcium Dynamics

*Sung Jin Park[1], Jaehoon Jeong[1], Young-Un Park[1], Kyung-Sun Park[2], Haeryun Lee[2], Namgyu Lee[1], Sung-Mo Kim[1], Keisuke Kuroda[3], Minh Dang Nguyen[4], Kozo Kaibuchi[3] and Sang Ki Park[1]**

[1]*Department of Life Sciences, Pohang University of Science and Technology, 790-784, Republic of Korea*

[2]*Division of Integrative Biosciences and Biotechnology, Pohang University of Science and Technology, 790-784, Republic of Korea*

[3]*Department of Cell Pharmacology, Nagoya University Graduate School of Medicine, Nagoya, Aichi 466-8550, Japan*

[4]*Hotchkiss Brain Institute, Departments of Clinical Neurosciences, Cell Biology and Anatomy, and Biochemistry and Molecular Biology, University of Calgary, Calgary, AB, Canada T2N 4N1*

** Correspondences should be addressed to SKP at skpark@postech.ac.kr.*

Supplementary Information

Supplementary Figure 1. Immunocytochemical analysis of the DISC1 mutant lacking amino acid residues 1-350 (DISC1$^{\Delta 1\text{-}350}$)

(a) Co-immunostaining of Flag-DISC1$^{\Delta 1\text{-}350}$ and GFP-ER (*i*) and a quantitative analysis of co-localization (*ii*) in HEK293 cells (n=6 for DISC1 WT, 4 for DISC1$^{\Delta 1\text{-}350}$). Scale bar represents 10 μm.

(b) Co-immunostaining of Flag-DISC1 and GFP-EXOC1 (*i*), Flag-DISC1$^{\Delta 1\text{-}350}$ and GFP-EXOC1 (*ii*), a quantitative analysis of co-localization (*iii*) in HEK293 cells (n=4 for DISC1 WT, 3 for DISC1$^{\Delta 1\text{-}350}$). Scale bar represents 10 μm.

Supplementary Figure 2. Multiple interaction interface of EXOC1 for DISC1

(a) Schematic mapped domains of EXOC1. (PH; pleckstrin homology, CC; coiled-coil, HB; helical bindles)

(b) Co-immunoprecipitation of GFP-EXOC1 fragments with Flag-DISC1 in HEK293 cells.

Supplementary Figure 3. Characterization of shRNA constructs specific for DISC1 and EXOC1

RNAs were acquired with the RNA preparation kit (SolGent). Complementary DNAs were produced from isolated RNAs with the Superscript III kit (Invitrogen), and the PCR bands were subjected to statistical analyses. Error bars represent means ±SEM. *; P<0.05, **; P<0.01 (two-tailed t-test)

(a) PCR bands of endogenous DISC1 (*i*) and Flag-DISC1 protein bands (*ii*) from control shRNA-, DISC1 shRNA1- or shRNA2-transfected HEK293 cells. DISC1 shRNA2 was mainly used for human DISC1 knockdown in HEK293 cells (n=9 for (*i*), 4 for (*ii*)).

(b) PCR bands of endogenous EXOC1 (*i*) and GFP-EXOC1 protein bands (*ii*) from control shRNA- or EXOC1 shRNA-transfected HEK293 cells (n=3 for (*i*), 4 for (*ii*)).

(c) PCR bands of endogenous mEXOC1 (*i*) and GFP-mEXOC1 protein bands (*ii)* from control shRNA- or mEXOC1 shRNA-transfected mouse neuroblastoma CAD cells (n=8 for (*i*), 3 for (*ii*)).

Supplementary Figure 4. PKA-mediated phosphorylation of IP$_3$R1 in EXOC1-knockdown

Phosphorylation of IP$_3$R1 in EXOC1 knockdown HEK293 cells. (n= 6). Error bars represent means ±SEM.

Statistical significance was analyzed by two-tailed *t*-test.

Supplementary Figure 5. Effects of enhanced PKA activity on IP3R1 phosphorylation by forskolin and ER calcium dynamics

(a) Up-regulation of IP3R1 phosphorylation upon treatment with 100 µM forskolin in HEK293 cells.

(b) ER calcium response curves under 50 µM ATP stimulation in 100 µM forskolin-treated HEK293 cells (*i*), and statistically analyzed average area (*ii*) and amplitude (*iii*) (n=80 for vehicle, 80 for forskolin).

Error bars represent means ±SEM. ***; *P*<0.001 (two-tailed *t*-test)

Supplementary Figure 6. IP3R-dependent ER calcium responses of DISC1 overexpression in EXOC1-knockdown cells and EXOC1 overexpression in DISC1-knockdown cells

(a) Rescue of abnormal IP3R-dependent ER calcium dynamics in EXOC1-knockdown cells. Effect of EXOC1 overexpression in DISC1-knockdown cells were analyzed in parallel, Average area (b) and amplitudes (c) of calcium response graph under 50 µM ATP stimulation were statistically analyzed (n= 79 for control shRNA, 26 for EXOC1 shRNA, 24 for DISC1 shRNA, 27 for EXOC1 shRNA+Flag-DISC1, 24 for EXOC1 shRNA+RFP-EXOC1). Error bars represent means ±SEM. *; *P*<0.05, **; *P*<0.01. ***; *P*<0.001 (two-tailed *t*-test)

Supplementary Figure 7. ER calcium responses upon blockade of mitochondrial calcium uptake

(a) IP3R-dependent ER calcium dynamics upon DISC1 or EXOC1 knockdown in HEK293 cells treated with ruthenium red (10 µM). Average area (b) and amplitudes (c) of the calcium response graph under 50 µM ATP were statistically analyzed (n= 44 for control shRNA, 47 for EXOC1 shRNA, 72 for DISC1 shRNA). Error bars represent means ±SEM. *; *P*<0.05, **; *P*<0.01. ***; *P*<0.001 (two-tailed *t*-test)

Supplementary Figure 8. No significant changes in ER basal calcium level and caffeine-induced ER calcium release in DISC1- or EXOC1-knockdown cells

(a) Co-localization between GFP-ER (green) and ER-aequorin (ER-AEQ, red) in CAD cells (*i*) and relative ER calcium responses detected by reconstituted ER-AEQ in mDISC1- and mEXOC1-knockdown CAD cells (*ii*). Amplitudes (*iii*) were statistically analyzed (n= 10 for control shRNA, 4 for mEXOC1 shRNA, 6 for mDISC1

shRNA).

(b) Caffeine-induced ER calcium dynamics in DISC1- or EXOC1-knockdown cells. Average area (*ii*) and amplitudes (*iii*) of the calcium response graph under 100 mM caffeine stimulation (*i*) were statistically analyzed (n= 16 for control shRNA, 25 for EXOC1 shRNA, 19 for DISC1 shRNA, 24 for DISC1 shRNA+EXOC1 shRNA).

Error bars represent means ±SEM. Statistical significance was analyzed by two-tailed *t*-test.

Supplementary Figure 9. Original gel (blot) data in main figures 1-3

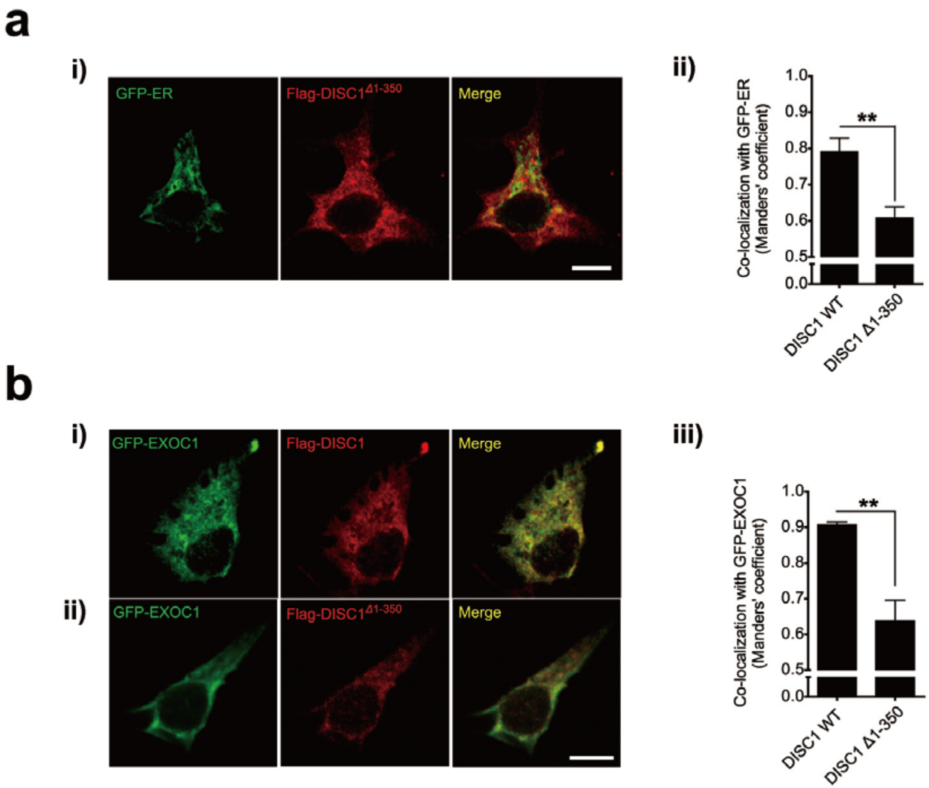
a
i)
GFP-ER
Flag-DISC1$^{Δ1-350}$
Merge
ii)
Co-localization with GFP-ER (Manders' coefficient)
1.0
0.9
0.8
0.7
0.6
0.5
0.0
**
DISC1 WT
DISC1 Δ1-350
b
i)
GFP-EXOC1
Flag-DISC1
Merge
ii)
GFP-EXOC1
Flag-DISC1$^{Δ1-350}$
Merge
iii)
Co-localization with GFP-EXOC1 (Manders' coefficient)
1.0
0.9
0.8
0.7
0.6
0.5
0.0
**
DISC1 WT
DISC1 Δ1-350

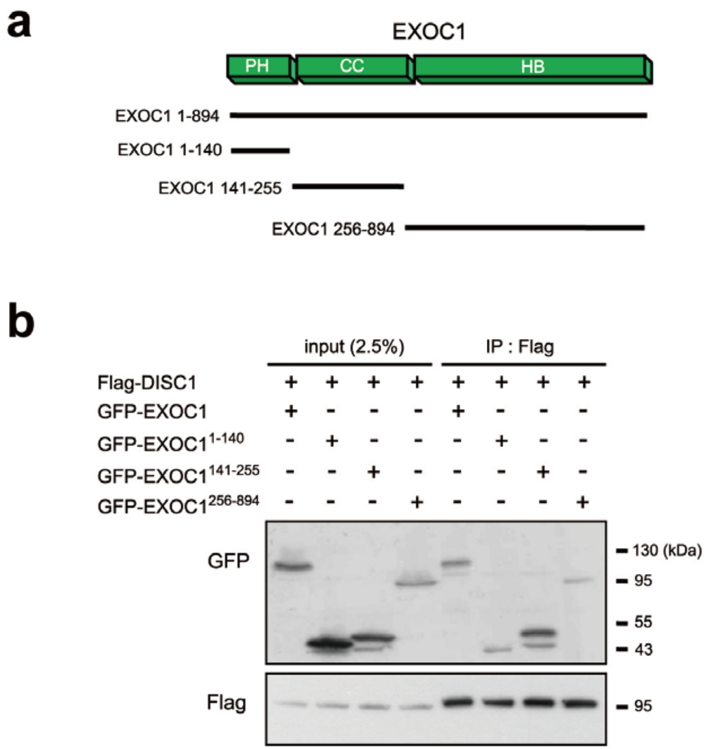
a
EXOC1
PH
CC
HB
EXOC1 1-894
EXOC1 1-140
EXOC1 141-255
EXOC1 256-894
b
input (2.5%)
IP : Flag
Flag-DISC1
GFP-EXOC1
GFP-EXOC1 1-140
GFP-EXOC1 141-255
GFP-EXOC1 256-894
GFP
Flag
130 (kDa)
95
55
43
95

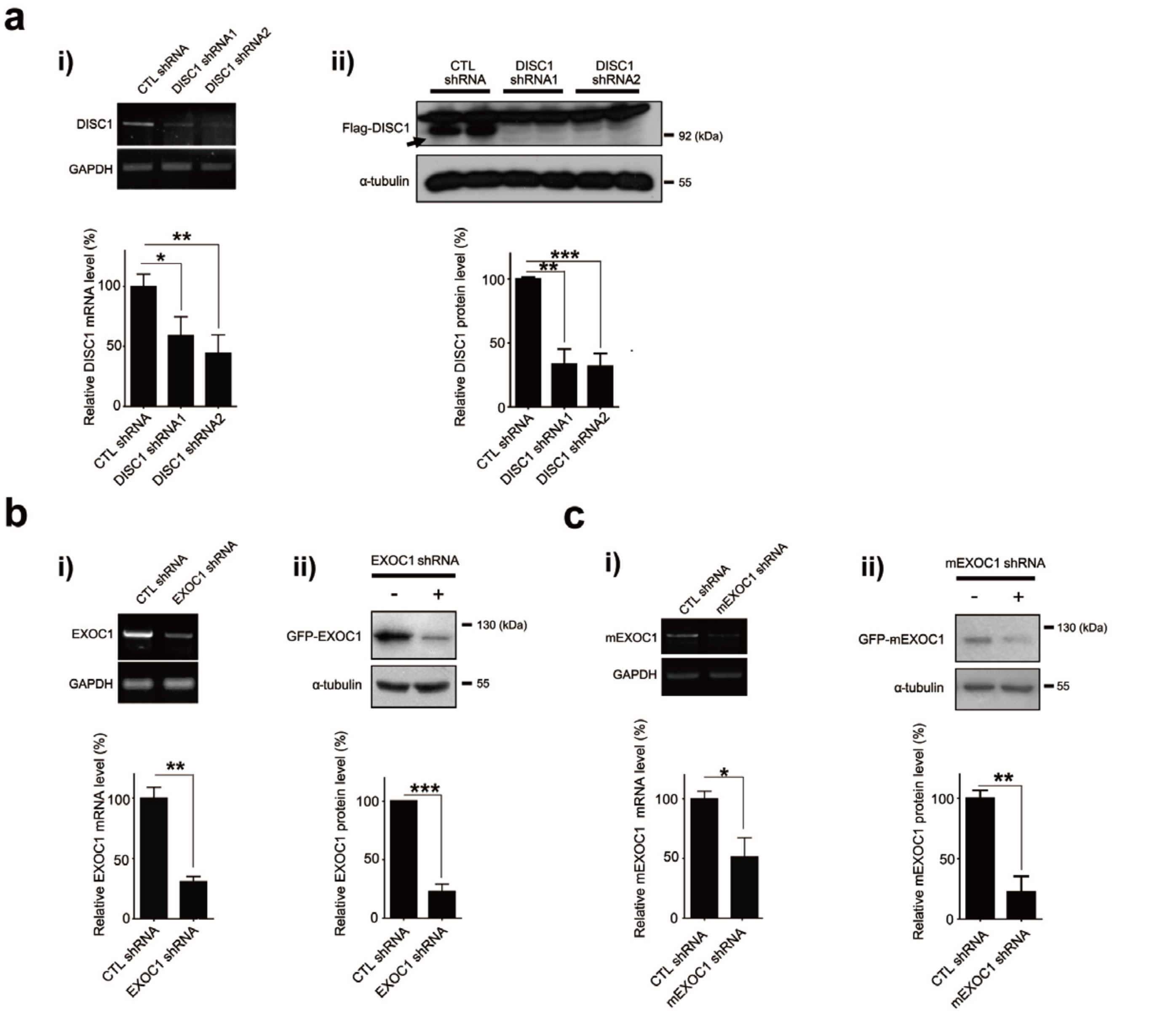

a
i)
CTL shRNA
DISC1 shRNA1
DISC1 shRNA2
DISC1
GAPDH
Relative DISC1 mRNA level (%)
ii)
CTL shRNA
DISC1 shRNA1
DISC1 shRNA2
Flag-DISC1
α-tubulin
92 (kDa)
55
Relative DISC1 protein level (%)
b
i)
CTL shRNA
EXOC1 shRNA
EXOC1
GAPDH
Relative EXOC1 mRNA level (%)
ii)
EXOC1 shRNA
GFP-EXOC1
α-tubulin
130 (kDa)
55
Relative EXOC1 protein level (%)
c
i)
CTL shRNA
mEXOC1 shRNA
mEXOC1
GAPDH
Relative mEXOC1 mRNA level (%)
ii)
mEXOC1 shRNA
GFP-mEXOC1
α-tubulin
130 (kDa)
55
Relative mEXOC1 protein level (%)

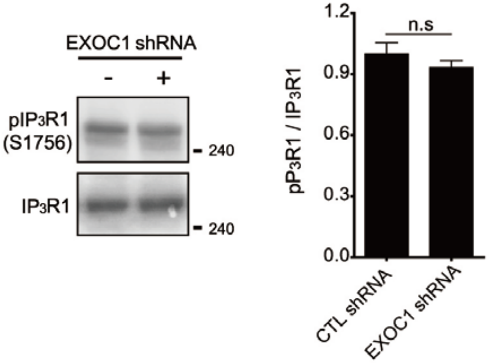
EXOC1 shRNA
-
+
pIP3R1
(S1756)
240
IP3R1
240
1.2
0.9
0.6
0.3
0.0
n.s
pP3R1 / IP3R1
CTL shRNA
EXOC1 shRNA

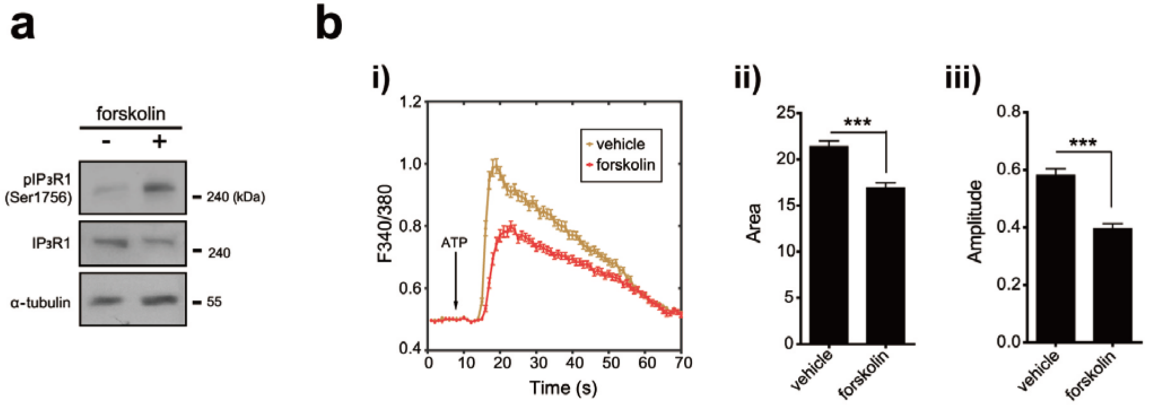

a
forskolin
-
+
pIP3R1
(Ser1756)
IP3R1
α-tubulin
240 (kDa)
240
55
b
i)
ii)
iii)
F340/380
ATP
vehicle
forskolin
Time (s)
Area
Amplitude
***
***
vehicle
forskolin
vehicle
forskolin

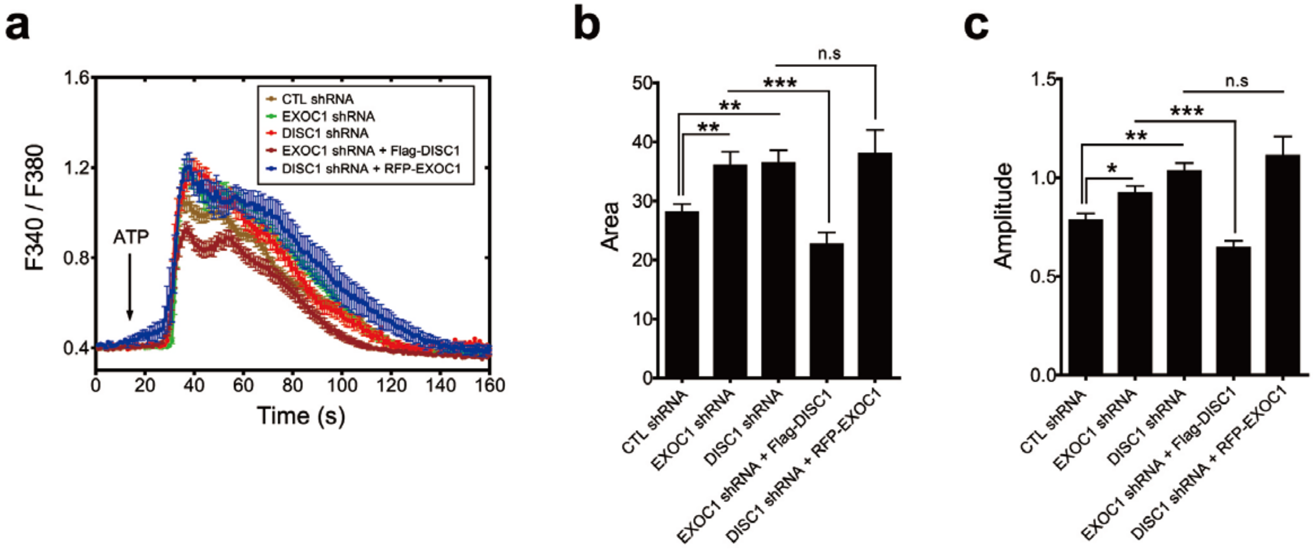
a
b
c
F340 / F380
Time (s)
ATP
CTL shRNA
EXOC1 shRNA
DISC1 shRNA
EXOC1 shRNA + Flag-DISC1
DISC1 shRNA + RFP-EXOC1
Area
Amplitude
n.s
***
**
*

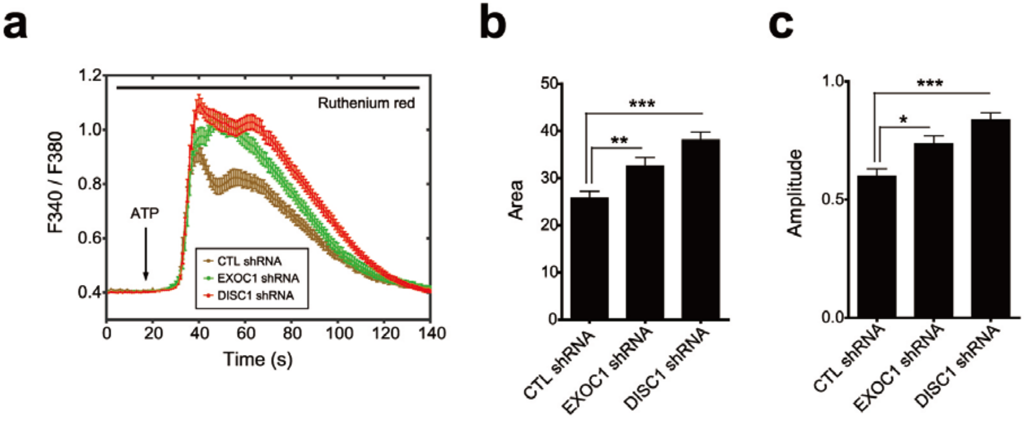
a
b
c
1.2
1.0
0.8
0.6
0.4
F340 / F380
Ruthenium red
ATP
CTL shRNA
EXOC1 shRNA
DISC1 shRNA
0
20
40
60
80
100
120
140
Time (s)
50
40
30
20
10
0
Area
***
**
CTL shRNA
EXOC1 shRNA
DISC1 shRNA
1.0
0.5
0.0
Amplitude
***
*
CTL shRNA
EXOC1 shRNA
DISC1 shRNA

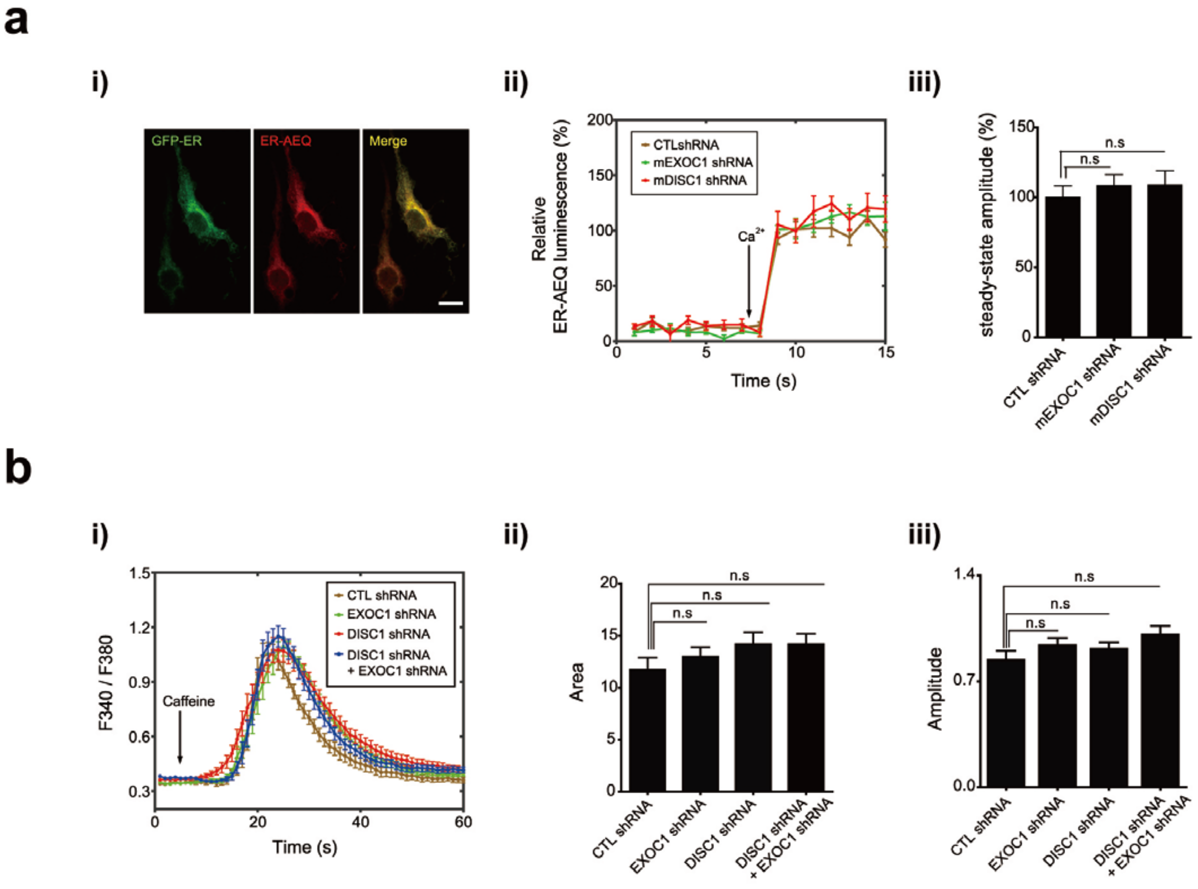

a
i)
GFP-ER
ER-AEQ
Merge
ii)
Relative ER-AEQ luminescence (%)
CTLshRNA
mEXOC1 shRNA
mDISC1 shRNA
Ca2+
Time (s)
iii)
steady-state amplitude (%)
n.s
n.s
CTL shRNA
mEXOC1 shRNA
mDISC1 shRNA
b
i)
F340 / F380
Caffeine
CTL shRNA
EXOC1 shRNA
DISC1 shRNA
DISC1 shRNA + EXOC1 shRNA
Time (s)
ii)
Area
n.s
n.s
n.s
CTL shRNA
EXOC1 shRNA
DISC1 shRNA
DISC1 shRNA + EXOC1 shRNA
iii)
Amplitude
n.s
n.s
n.s
CTL shRNA
EXOC1 shRNA
DISC1 shRNA
DISC1 shRNA + EXOC1 shRNA

Figure 1aii

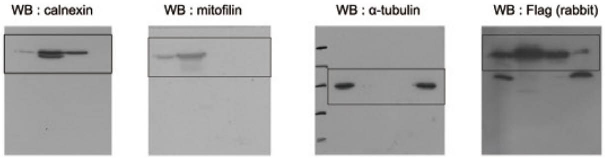

Figure 1aiii

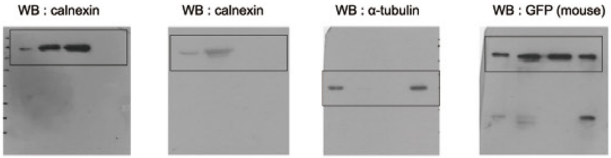

Figure 1aiii

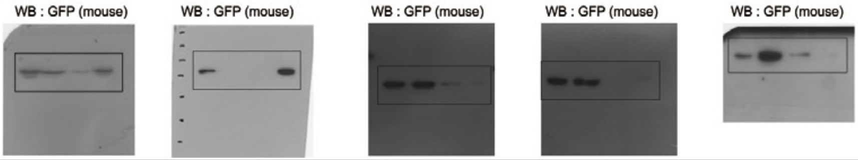

Figure 2a

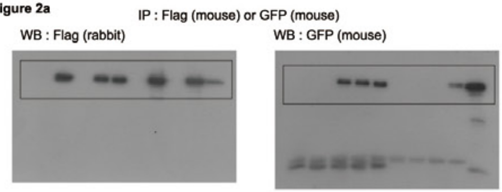

Figure 2c

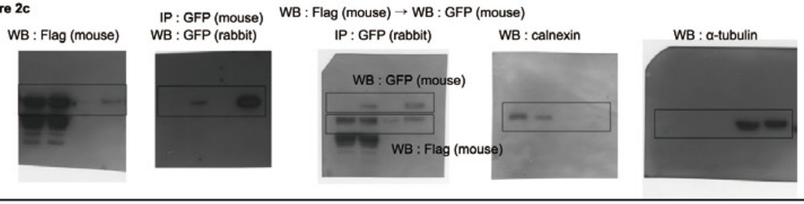

Figure 2d

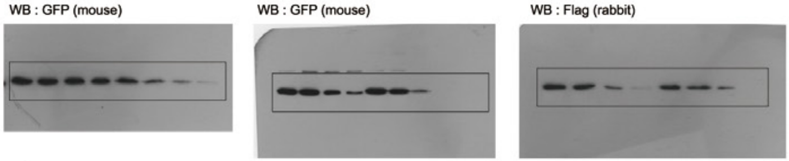

Figure 3a

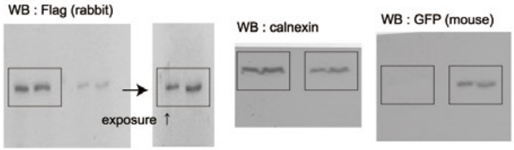

Figure 3b

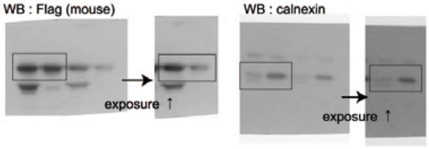

Figure 3c

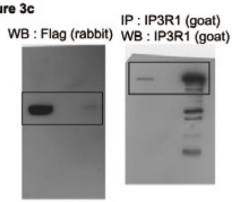

Figure 3d

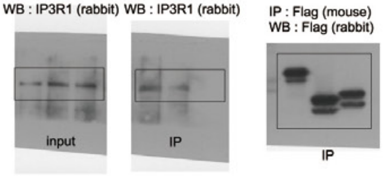

Figure 3e

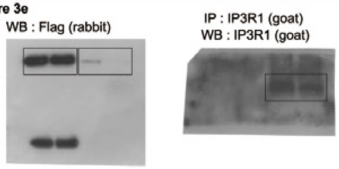

Figure 3f

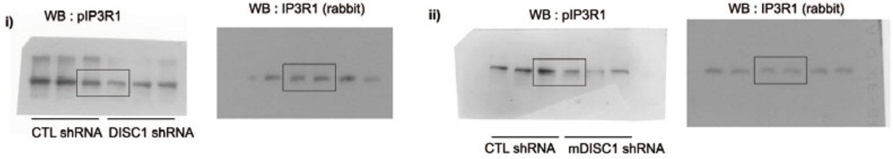

Supplement: Supplementary Information [file srep08694-s1.pdf]
